# Tumor CTR1 Expression and Systemic Copper Dynamics Converge on a Copper Axis in High-Grade Triple-Negative Breast Cancer

**DOI:** 10.1158/2767-9764.CRC-26-0036

**Published:** 2026-06-30

**Authors:** Vinit Shanbhag, Nikita S. Gudekar, Muhammad Yasir, Kristyn Conrad, Samuel Anakpeba-Dinguyella, Parshad Suthar, Praveen Rao, Michael Petris, Linda T. Vahdat, Christos Papageorgiou

**Affiliations:** 1 Department of Biochemistry, Life Sciences Center, University of Missouri, Columbia, Missouri.; 2 Ellis Fischel Cancer Center, University of Missouri, Columbia, Missouri.; 3 Department of Electrical Engineering and Computer Science, College of Engineering, University of Missouri, Columbia, Missouri.; 4 Department of Ophthalmology, MU School of Medicine, University of Missouri, Columbia, Missouri.; 5 Dartmouth Cancer Center, Dartmouth Hitchcock Medical Center, Geisel School of Medicine at Dartmouth, Lebanon, New Hampshire.

## Abstract

Copper (Cu) metabolism represents a unique vulnerability in cancer. CTR1 (*SLC31A1*) mediates cellular Cu uptake, but its relationship with tumor aggressiveness and neoadjuvant response is unclear. We tested whether tumor *SLC31A1* expression and systemic Cu dynamics reflect a coordinated tumor-systemic Cu axis in breast cancer. Baseline tumor *SLC31A1* gene expression was analyzed in a retrospective cohort of 1,632 neoadjuvant-treated patients with breast cancer, who were subsequently stratified by pathologic response across molecular subtypes and tumor grades. These findings were extended to an exploratory prospective neoadjuvant cohort, in which ΔCu, defined as the posttreatment to pretreatment change in serum Cu, was assessed by subtype, grade, and response, whereas baseline serum Cu was examined by tumor size. Baseline *SLC31A1* expression was higher in triple-negative breast cancer (TNBC) nonresponders than in responders (*P* = 0.0021), particularly in grade 3 tumors (*P* = 0.0035), with no differences in luminal subtypes. In the prospective cohort, ΔCu changes were most pronounced in TNBC and were strongly grade-dependent: all grade 3 TNBCs showed posttherapy Cu elevation, whereas all grade 2 TNBCs showed decreases (*P* = 0.034). The relapsed TNBC nonresponder exhibited persistently positive ΔCu, whereas nonresponders from other subtypes showed near-zero or negative changes (*P* = 0.011). Baseline serum Cu was higher in patients with smaller (T1) versus larger (T2–T3) tumors (*P* = 0.033). Elevated baseline tumor *SLC31A1* expression and posttherapy systemic Cu increases converge in high-grade TNBC, indicating a potential coordinated Cu mobilization program linked to aggressive biology and neoadjuvant resistance.

**Significance:**

*SLC31A1* expression was evaluated in a large retrospective cohort, providing robust evidence that elevated baseline *SLC31A1* in high-grade TNBC associates with nonresponse. ΔCu dynamics were assessed in a prospective cohort. Despite the limited size, the consistent ΔCu increase in grade 3 TNBC and a relapsing nonresponder supports a biologically meaningful resistance signal. Together, these datasets define a coordinated Cu axis warranting prospective validation and early Cu-targeted intervention.

## Introduction

Breast cancer remains a leading cause of cancer-related morbidity and mortality worldwide, with heterogeneous outcomes across molecular subtypes. Despite advances in targeted and immune-based therapies, there remains an unmet need for approaches that capture tumor biology and treatment response in real time, particularly in aggressive disease.

Copper (Cu) is an essential nutrient required for energy production, antioxidant defense, and connective tissue maturation, yet its metabolism has emerged as a unique vulnerability in cancer (1). Cellular Cu uptake is primarily mediated by the high-affinity transporter CTR1 (*SLC31A1*), the principal gateway for Cu entry and a key regulator of intracellular Cu availability (2, 3). Global *Ctr1* deletion in mice causes embryonic lethality (4), and intestinal loss results in systemic Cu deficiency (5), underscoring its physiologic importance. CTR1-driven Cu uptake supports several oncogenic programs. Cu-dependent activation of the MAPK-ERK pathway and ULK1/2 signaling links CTR1-mediated Cu transport to cell growth, autophagy, and tumor progression (6, 7).

Elevated serum Cu has been associated with adverse prognosis, and intracellular Cu availability supports multiple tumor-promoting pathways (1). These include angiogenesis through VEGF signaling (8), mitochondrial respiration via cytochrome c oxidase (9, 10), antioxidant defense through superoxide dismutase (11, 12), extracellular matrix (ECM) remodeling through lysyl oxidases (13–15), and regulation of signaling kinases such as MEK1 and ULK1 (6, 7). In an effort to counteract these oncogenic effects of Cu, a phase II clinical study of systemic Cu depletion with tetrathiomolybdate in high-risk patients with breast cancer demonstrated feasibility and suggested benefit in triple-negative breast cancer (TNBC; ref. 16).

Despite these insights, how Cu mobilization and CTR1-mediated uptake are regulated across tumor grades, aggressiveness, and treatment contexts remains poorly understood. Most prior studies have examined static baseline Cu levels rather than dynamic changes in Cu transport or systemic Cu availability. To address this gap, we performed a large retrospective analysis of baseline tumor *SLC31A1* gene expression, stratifying patients by subsequent pathologic response to neoadjuvant chemotherapy. These findings were extended to an exploratory prospective study measuring paired pretreatment and posttreatment serum Cu levels (ΔCu) in the neoadjuvant setting. This integrative approach enables parallel evaluation of tumor Cu demand and systemic Cu mobilization, providing exploratory insight into a coordinated tumor-systemic Cu axis in breast cancer.

## Materials and Methods

### Study design

**Figure 1.**
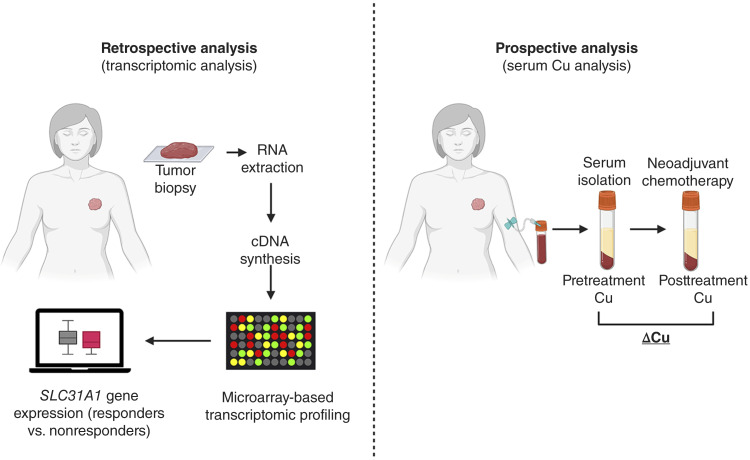
Overview of retrospective and prospective study design. The retrospective arm compared baseline tumor *SLC31A1* gene expression in patients stratified by subsequent pathologic response to neoadjuvant therapy, and the prospective arm measured serum Cu changes (ΔCu) before and after neoadjuvant therapy.

This study incorporated a retrospective gene expression analysis and an exploratory prospective observational cohort to examine Cu biology in breast cancer (Fig. 1). The retrospective arm evaluated baseline tumor *SLC31A1* gene expression in public datasets, whereas the prospective arm measured serum Cu dynamics (ΔCu) during neoadjuvant therapy. Both datasets were analyzed independently and then compared with identify hypothesis-generating convergent trends between tissue gene expression and systemic Cu changes.

### Ethics approval and consent to participate

The prospective observational component of this study involving human participants was reviewed and approved by the University of Missouri Institutional Review Board (IRB #2026923). Written informed consent was obtained from all participants prior to enrollment. All clinical samples and associated data were coded, deidentified, and handled in accordance with institutional policies and Health Insurance Portability and Accountability Act regulations. The study was conducted in accordance with recognized ethical guidelines for human subjects research, including the Declaration of Helsinki and applicable US Common Rule requirements.

The retrospective gene expression analyses were conducted using ROCplot.org, which integrates publicly available, deidentified breast cancer transcriptomic datasets. Analysis of these public datasets did not require additional IRB approval and was performed in accordance with the data access and usage policies of the original data sources.

### Retrospective analysis of *SLC31A1* gene expression

Baseline tumor gene expression of *SLC31A1* (encoding CTR1) was analyzed using the ROCplot.org platform (17), which integrates transcriptomic and treatment–response data from 36 publicly available breast cancer datasets. The analysis focused on patients with pathologic response data (*n* = 1,632) following neoadjuvant chemotherapy. Subgroup analyses were performed by molecular subtype (triple-negative, luminal A, and luminal B) and by tumor grade within the TNBC cohort. Differences in *SLC31A1* gene expression between responders and nonresponders were assessed using the Mann–Whitney U test. Discriminatory performance was summarized using the area under the receiver operating characteristic curve (AUC). Supplemental survival analysis for *SLC31A1* in grade 3 TNBC was performed using KMplotter, and pan-cancer tumor versus normal *SLC31A1* gene expression analysis was performed using GEPIA based on The Cancer Genome Atlas (TCGA) datasets.

### Subtyping and clinical classification

Breast cancer subtypes were defined by estrogen receptor (ER), progesterone receptor (PR), and human epidermal growth factor receptor 2 (HER2) status assessed by immunohistochemistry (IHC) and confirmed by fluorescence *in situ* hybridization (FISH). TNBC was defined as ER <1%, PR <1%, and HER2-negative by IHC or FISH. Tumor grade and pathologic stage were from final pathology reports. Pathologic response to neoadjuvant therapy was classified as response or nonresponse based on clinical and pathologic assessment.

### Serum collection, Cu measurement, and ceruloplasmin activity assays

As serum Cu is not materially affected by the time of day or postprandial interval (18, 19), patients were not required to have fasted prior to blood draws, thus minimizing disruption of standard clinical workflow in the neoadjuvant setting (20). Peripheral blood was collected by venipuncture at three time points: diagnosis (before therapy), completion of neoadjuvant chemotherapy, and biopsy-confirmed relapse. Serum was separated and stored at −80°C. Total Cu was quantified by inductively coupled plasma mass spectrometry (Agilent 8900 ICP-QQQ) using standard trace-metal workflows. Ceruloplasmin (CP) activity was measured in baseline serum samples from all patients in the prospective cohort and from healthy volunteers used as a reference group, as well as in paired before and after treatment serum samples from patients with breast cancer, using a previously described o-dianisidine–based colorimetric oxidase assay protocol (21), and is reported in units per liter (U/L).

### Clinical variables captured

Clinical variables were collected as part of routine clinical care, and research protocols and are reported in deidentified form. Treatment followed subtype-specific national guidelines.

### Primary exposure

ΔCu (before–after treatment serum Cu, μg/kg) represented the primary exposure. A positive value reflects higher Cu after therapy. The first posttherapy sample was used for analysis; later follow-up samples, including the relapse case, were summarized descriptively.

### Statistical analysis (prospective cohort)

Data are summarized as medians with interquartile ranges (IQR); means, minima, and maxima are reported where informative. Associations between ΔCu and clinicopathologic variables were examined using nonparametric methods. Correlations were evaluated with Spearman’s rank (ρ) and two-sided *P* values. Group comparisons used Mann–Whitney U tests; comparisons across multiple subtypes used Kruskal–Wallis tests. Analyses were performed in Python (v3.x) with the pandas library for data handling, NumPy (RRID: SCR_008633) for numerical computation, and SciPy (RRID: SCR_008058; scipy.stats) for statistical testing. Figures and graphs were generated in GraphPad Prism (v10; RRID: SCR_002798).

## Results

### Patient characteristics

A total of 21 patients with breast cancer were included in the exploratory prospective cohort. The cohort included patients ages 28 to 74 years and comprised premenopausal, perimenopausal, and postmenopausal subjects. Demographic characteristics are summarized in Table 1 and are presented in deidentified form in accordance with data-sharing requirements. Clinical variables were evaluated in relation to serum Cu dynamics, tumor subtype, grade, and treatment response and are analyzed in subsequent sections.

**Table 1.** Demographic and clinical characteristics of patients with breast cancer (*N* = 21).

Patient ID	Age (years)	BMI	Race	Ethnicity	Gender	Menopause	Diabetes
BCP-001	63	31.9	White	Non-Hispanic	F	Postmenopausal	Yes
BCP-002	62	34.9	White	Non-Hispanic	F	Postmenopausal	Prediabetic
BCP-003	37	38.8	White	Non-Hispanic	F	Premenopausal	No
BCP-004	28	35.7	Black	Non-Hispanic	F	Premenopausal	No
BCP-005	64	21.3	White	Non-Hispanic	F	Postmenopausal	No
BCP-006	37	29.2	White	Non-Hispanic	F	Premenopausal	No
BCP-007	54	28.52	White	Non-Hispanic	F	Perimenopausal	No
BCP-008	52	25.9	White	Non-Hispanic	F	Premenopausal	No
BCP-009	36	26	White	Non-Hispanic	F	Premenopausal	No
BCP-010	74	25.5	Asian	Non-Hispanic	F	Postmenopausal	No
BCP-011	43	21.5	White	Non-Hispanic	F	Premenopausal	No
BCP-012	61	37.9	White	Non-Hispanic	F	Postmenopausal	Yes
BCP-013	36	46.3	Black	Non-Hispanic	F	Premenopausal	Yes
BCP-014	55	40	White	Non-Hispanic	F	Perimenopausal	No
BCP-015	70	30.6	White	Non-Hispanic	F	Postmenopausal	No
BCP-016	71	24.6	White	Non-Hispanic	F	Postmenopausal	No
BCP-017	56	22.7	White	Non-Hispanic	F	Perimenopausal	No
BCP-018	62	24.6	White	Non-Hispanic	F	Postmenopausal	No
BCP-019	52	31.4	White	Non-Hispanic	F	Perimenopausal	No
BCP-020	48	38.4	White	Non-Hispanic	F	Postmenopausal	Yes
BCP-021	73	45.9	White	Non-Hispanic	M	N/A	No

Values represent individual patient data. Patient IDs shown are anonymized for presentation and do not correspond to original identifiers.

Abbreviations: BMI, body mass index; N/A, not applicable.

### Retrospective analysis of *SLC31A1* expression and pathologic response

**Figure 2.**
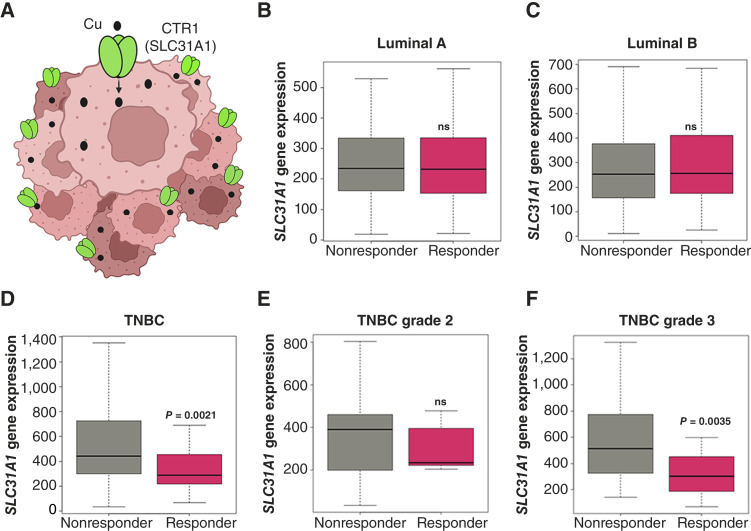
CTR1 protein function and *SLC31A1* expression by pathologic response. **A,** Illustration of CTR1 on the tumor cell surface mediating Cu uptake. **B–D,** Baseline tumor *SLC31A1* transcript levels stratified by subsequent pathologic response across luminal A, luminal B, and TNBC. **E** and **F,***SLC31A1* expression in grade 2 and grade 3 TNBCs stratified by pathologic response. ns, not significant.

The integrated study design is shown in Fig. 1. To evaluate whether baseline tumor Cu import capacity associates with response to neoadjuvant chemotherapy, baseline *SLC31A1* gene expression was analyzed in 1,632 patients with breast cancer with available pathologic response data (17). Figure 2A illustrates CTR1 protein localization on tumor cells and its role in facilitating Cu uptake.

Across molecular subtypes, no significant differences in *SLC31A1* gene expression were observed in luminal A tumors (*n* = 341 nonresponders vs. 134 responders, *P* = 0.44, AUC = 0.509; Fig. 2B) or luminal B tumors (*n* = 372 nonresponders vs. 119 responders, *P* = 0.36, AUC = 0.522; Fig. 2C; Table 2). In contrast, *SLC31A1* gene expression was significantly elevated in TNBC nonresponders (*n* = 277) compared with responders (*n* = 196, *P* = 0.0021; Fig. 2D; Table 2), with a corresponding AUC of 0.682. Within TNBC, *SLC31A1* gene expression also varied by histologic grade. In grade 2 tumors, no significant difference was observed between nonresponders and responders (*n* = 42 vs. 13, *P* = 0.54, AUC = 0.576; Fig. 2E; Table 2). In contrast, grade 3 tumors showed significantly higher *SLC31A1* expression in nonresponders (*n* = 172) than in responders (*n* = 89, *P* = 0.0035; Fig. 2F; Table 2), with an AUC of 0.717.

**Table 2.** Sample distribution and statistical comparison for *SLC31A1* analysis across breast cancer subtypes.

Subtype	Nonresponders (*n*)	Responders (*n*)	*P* value (Mann–Whitney U)
TNBC	277	196	0.0021
TNBC grade 3	172	89	0.0035
TNBC grade 2	42	13	0.54 (not significant)
Luminal A	341	134	Not significant
Luminal B	372	119	Not significant

Together, these findings indicate that elevated baseline *SLC31A1* gene expression associates with chemotherapy nonresponse and higher tumor grade specifically in TNBC, providing the rationale for examining dynamic systemic Cu changes in the prospective cohort.

### Distribution of ΔCu across breast cancer molecular subtypes

**Figure 3.**
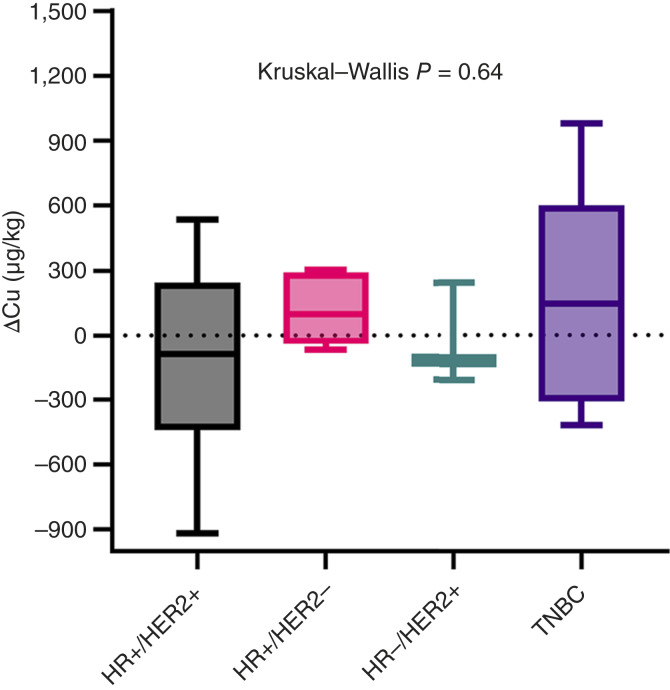
ΔCu levels in patients with breast cancer by molecular subtype. Box plots show the distribution of ΔCu values (after treatment − before treatment) across subtypes. The horizontal line within each box represents the median, box edges represent the IQR, and whiskers indicate the minimum and maximum values. The Kruskal–Wallis *P* value is shown on the plot.

To explore the dynamic behavior of serum Cu following treatment, ΔCu, defined as the change in serum Cu concentration from before to after treatment, was quantified across breast cancer molecular subtypes, representing a novel and dynamic metric introduced in this cohort. Median ΔCu values differed by subtype, with TNBC (*n* = 6) showing the highest median increase of 123 μg/kg (IQR, 433.8 μg/kg) followed by hormone receptor (HR)+/HER2− (*n* = 6) at 98.5 μg/kg (IQR, 263.3 μg/kg), whereas HR+/HER2+ (*n* = 6) and HR−/HER2+ (*n* = 3) exhibited median decreases of −89.5 μg/kg (IQR, 407.5 μg/kg) and −119 μg/kg (IQR, 225.5 μg/kg), respectively (Fig. 3; Table 3). Although overall distributions did not differ significantly among subtypes (Kruskal–Wallis H = 1.71, *P* = 0.64), TNBC displayed the broadest range of values (−416 to +978 μg/kg) and a trend toward higher ΔCu compared with other groups. Because CP is the major circulating Cu carrier and an acute phase reactant (22, 23), and its oxidase activity is widely used as a standard functional readout of Cu-loaded CP, we next examined whether the observed serum Cu patterns could be explained by CP activity. Baseline CP activity did not show a uniform elevation across breast cancer subtypes relative to healthy volunteers (Supplementary Fig. S1; Supplementary Table S1), and baseline serum Cu did not show a tight relationship with CP activity across the cohort (Supplementary Fig. S2). In paired analyses, therapy associated changes in serum Cu (ΔCu) were not consistently mirrored by corresponding changes in CP activity (ΔCP; Supplementary Fig. S3; Supplementary Table S2), suggesting that CP-associated inflammation is not a primary contributor to the observed Cu dynamics.

**Table 3.** ΔCu levels in patients with breast cancer by molecular subtype (*N* = 21).

Subtype	*n*	Median ΔCu μg/kg	Mean ΔCu μg/kg	IQR	Min	Max
HR+/HER2+	6	−89.5	−116.8	407.5	−919	534
HR+/HER2−	6	98.5	114.5	263.3	−72	304
HR−/HER2+	3	−119	−28.7	225.5	−209	242
TNBC	6	123	126.2	433.8	−416	978

### Distinct ΔCu pattern in the TNBC relapse case

**Figure 4.**
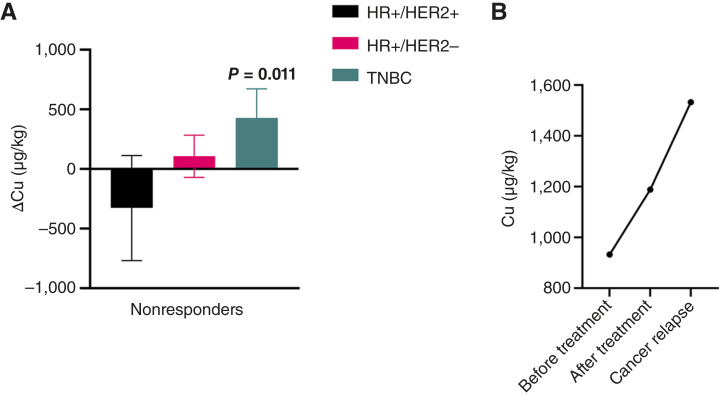
ΔCu and relapse in breast cancer nonresponders. **A,** Distribution of ΔCu values in nonresponders across molecular subtypes. **B,** Serum Cu trajectory of the single patient with TNBC who relapsed, showing changes from before treatment to after treatment and relapse follow-up.

Because treatment resistance may be associated with distinct Cu dynamics, ΔCu values were examined in nonresponders across subtypes (Fig. 4). In HR+/HER2+ (*n* = 4) and HR+/HER2− (*n* = 5) subtypes, most nonresponders showed near-zero or negative ΔCu, whereas in TNBC, the single nonresponder, who subsequently relapsed, demonstrated consistently positive ΔCu values at both follow-up time points, which were specifically measured to track changes after treatment and at cancer relapse (Fig. 4A). This difference among nonresponders reached statistical significance (Spearman ρ = 0.73; *P* = 0.011). In Fig. 4B, longitudinal serum Cu measurements for this single TNBC relapse case are presented as a descriptive example, showing progressive increases at each time point: from 932 μg/kg before treatment to 1,188 μg/kg at first follow-up and 1,533 μg/kg at cancer relapse.

### Tumor grade correlates with ΔCu in TNBC

**Figure 5.**
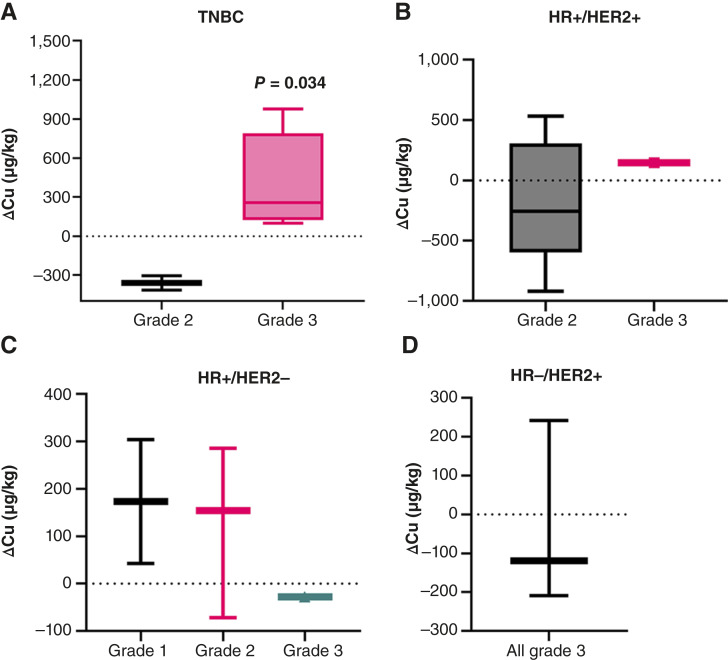
ΔCu levels stratified by tumor grade within molecular subtypes. Box plots display ΔCu values (after treatment − before treatment) by tumor grade within each molecular subtype: (**A**) TNBC, (**B**) HR+/HER2+, (**C**) HR+/HER2−, and (**D**) HR−/HER2+. The horizontal line within each box represents the median, box edges indicate the IQR, and whiskers extend to the minimum and maximum values.

We next assessed ΔCu by tumor grade within each subtype, a recognized marker for tumor aggressiveness (Fig. 5). In the TNBC group, a striking pattern emerged: all the patients harboring grade 3 tumors (*n* = 4) exhibited positive ΔCu values, whereas all the patients harboring grade 2 tumors (*n* = 2) displayed negative ΔCu values (Fig. 5A). This association was statistically significant (Spearman ρ = 0.79; *P* = 0.034). Conversely, no consistent or significant correlations were identified between tumor grade and ΔCu in the HR+/HER2+, HR+/HER2−, or HR−/HER2+ subtypes (Fig. 5B–D). These findings highlight a grade-related Cu dynamic unique to TNBC in this cohort.

### Inverse association between tumor size and pretreatment serum Cu

**Figure 6.**
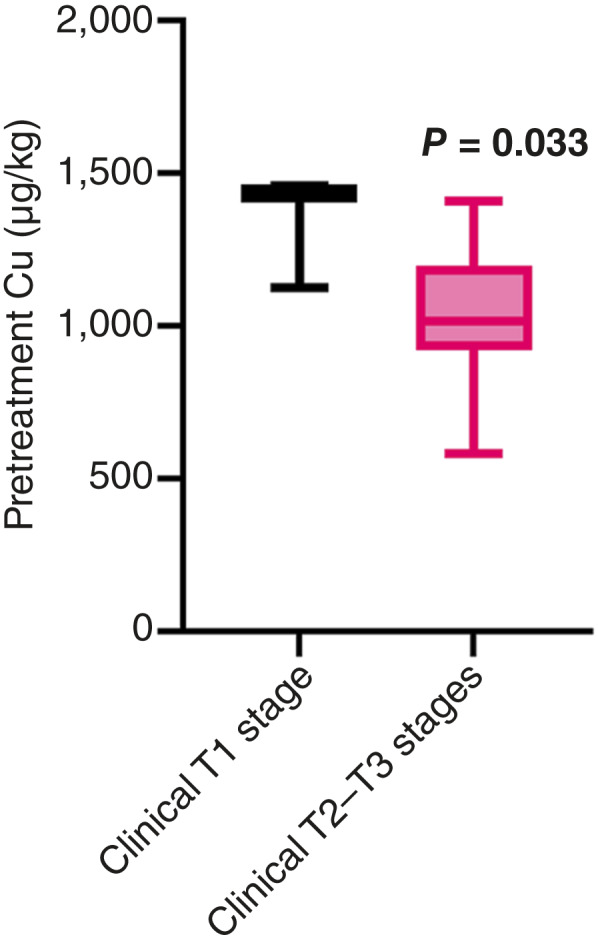
Serum Cu levels by clinical T stage. Box plots show serum Cu concentrations (μg/kg) for patients with stage T1 (*n* = 3) and stages T2–T3 (*n* = 16) breast tumors. The horizontal line within each box indicates the median, box edges represent the IQR, and whiskers denote the minimum and maximum values.

We next examined whether pretreatment baseline serum Cu levels were associated with clinical tumor size at diagnosis (Fig. 6). Prior preclinical studies have reported higher intratumoral Cu concentrations in smaller tumors (10), and thus we evaluated whether this relationship extended to systemic serum Cu. Female patients were grouped by clinical tumor stage as having T1 tumors (≤2 cm; *n* = 3) or T2 to T3 tumors (>2 cm; *n* = 16). Median pretreatment serum Cu levels were significantly higher in patients with smaller T1 tumors compared with those harboring larger T2 to T3 tumors [1,435 μg/kg (IQR, 166 μg/kg) vs. 1,015 μg/kg (IQR, 268 μg/kg); Mann–Whitney U = 43, *P* = 0.033].

## Discussion

Cu supports multiple metabolic and signaling processes essential to cancer progression, including mitochondrial respiration, redox maintenance, angiogenesis, and ECM remodeling (1). Clinical studies of systemic Cu chelation in high-risk TNBC suggest that Cu metabolism can be therapeutically targeted in this subtype (16). However, key questions remain: Why does TNBC show such a strong association with Cu biology? Is Cu linked to tumor aggressiveness and resistance to neoadjuvant chemotherapy? Can Cu-associated biomarkers guide personalized treatment or Cu chelation strategies?

To begin addressing these questions, we conducted the first integrated analysis that compared paired serum Cu measurements from an exploratory prospective neoadjuvant cohort with retrospective tumor *SLC31A1* gene expression linked to neoadjuvant chemotherapy response. This complementary design enabled parallel assessment of tumor Cu transporter biology and dynamic systemic Cu changes (ΔCu) during treatment, an aspect of Cu biology not previously evaluated in patients with breast cancer.

A consistent and clinically meaningful pattern emerged across both datasets: high-grade TNBC engages a coordinated Cu axis. In the retrospective analysis of 1,632 patients, baseline *SLC31A1* gene expression was significantly elevated only in grade 3 TNBC nonresponders, with no differences in grade 2 TNBC or luminal subtypes. In the prospective cohort, ΔCu behavior mirrored this specificity: all grade 3 TNBCs demonstrated positive ΔCu, whereas all grade 2 TNBCs showed negative ΔCu, and nonresponders from other subtypes tended to maintain or reduce systemic Cu levels. The only TNBC nonresponder, who later relapsed, displayed persistently rising ΔCu across the sampling timeline. Importantly, serum Cu changes were not consistently associated with CP activity, suggesting that they are not solely driven by CP-linked inflammatory responses. Taken together, these findings suggest that Cu metabolism is tightly linked to both tumor aggressiveness and treatment resistance in high-grade TNBC.

Several biologically plausible mechanisms may explain this pattern. Cu is required for enzymes and signaling mediators such as cytochrome c oxidase, superoxide dismutase, the lysyl oxidase (LOX/LOXL1–4) family, and kinases, including MEK1 and ULK1/2. These Cu-dependent activities support several processes fundamental to cancer cell survival and progression (1). Increased Cu import through CTR1 could enhance the activity of these or other Cu-dependent pathways, contributing to metabolic fitness, redox stability, autophagy regulation, and ECM remodeling, processes that may collectively promote aggressive tumor behavior and could attenuate chemotherapy efficacy. The LOX family is particularly relevant: LOX-mediated collagen cross-linking has been implicated in chemotherapy resistance in TNBC, and LOX inhibition can resensitize resistant models (24). Increased Cu availability may enhance LOX activity and ECM-mediated survival pathways, providing a plausible mechanism linking Cu metabolism to treatment resistance in high-grade TNBC.

We also observed that patients with smaller primary tumors had higher pretreatment serum Cu, aligning with the hypothesis that biologically active, early-stage tumors may trigger Cu mobilization from Cu-rich sources such as the liver to meet angiogenic and metabolic demands. This pattern parallels preclinical findings of elevated intratumoral Cu in smaller tumors (10). If validated, pretreatment Cu levels and ΔCu trajectories could serve as stage- or biology-informed biomarkers.

From a translational perspective, the convergence of tumor *SLC31A1* expression and ΔCu dynamics defines a hypothesis-generating biomarker framework for high-grade TNBC. Although these observations do not establish predictive utility, they support the presence of a biologically coherent Cu axis associated with aggressive disease and warrant future prospective validation. If confirmed, this integrated Cu signature may help identify patients with Cu-dependent and treatment-resistant biology and could complement existing clinicopathologic tools for risk stratification in the neoadjuvant setting.

Because tumor *SLC31A1* expression and systemic Cu measurements from our institutional cohort were derived from independent datasets, this convergence is interpreted as biological rather than causal. Although the exploratory prospective cohort size is modest, the reproducible, subtype- and grade-specific patterns observed across two independent datasets provide a strong basis for further investigation. Future studies should expand these observations in larger cohorts and incorporate ΔCu, ΔCP activity, and longitudinal Cu response metrics alongside tumor profiling of *SLC31A1* gene expression, Cu-dependent enzymes, and Cu-regulated signaling pathways to refine patient selection and clarify mechanisms linking Cu metabolism to neoadjuvant chemotherapy resistance. Consistent with this disease context, the supplemental KMplotter survival analysis showed that higher *SLC31A1* expression was associated with reduced survival in patients with grade 3 TNBC (Supplementary Fig. S4). To place these findings in a broader oncologic context, supplemental TCGA dataset analysis showed that *SLC31A1* gene expression is elevated in several tumor types relative to normal tissues (Supplementary Fig. S5A–S5H), supporting the broader relevance of CTR1 in cancer biology. Extending integrated analyses of tumor *SLC31A1* expression and systemic Cu dynamics across additional cancer types will be an important direction for future investigation.

In summary, this integrated analysis provides the initial clinical evidence consistent with a coordinated tumor-systemic Cu program in high-grade TNBC, characterized by elevated *SLC31A1* gene expression and increased ΔCu during neoadjuvant therapy. These hypothesis-generating findings strengthen the emerging link between Cu metabolism, tumor aggressiveness, and treatment resistance and provide a rationale for future studies evaluating Cu-related biomarkers in personalized therapeutic strategies and clinical trial designs.

## Supplementary Material

Figure S1 Baseline ceruloplasmin activity was measured across breast cancer molecular subtypes and healthy volunteers.

Figure S2 This figure shows the relationship between baseline serum copper concentration and ceruloplasmin activity across breast cancer subtypes and healthy volunteers.

Figure S3 This figure compares therapy-associated changes in serum copper and ceruloplasmin activity across breast cancer molecular subtypes.

Figure S4 This figure shows Kaplan–Meier survival analysis of grade 3 triple-negative breast cancer patients stratified by SLC31A1 expression.

Figure S5 This figure shows pan-cancer SLC31A1 expression in tumor versus normal tissues using TCGA data accessed through GEPIA.

Table S1 This table lists baseline serum copper and ceruloplasmin activity values for healthy volunteers and breast cancer patients grouped by molecular subtype.

Table S2 This table lists therapy-associated changes in serum copper and ceruloplasmin activity for breast cancer patients grouped by molecular subtype.

## Data Availability

The prospective clinical data supporting the findings of this study are not publicly available due to patient privacy and ethical considerations but may be made available from the corresponding author upon reasonable request and with appropriate institutional approvals. Publicly available gene expression datasets analyzed in this study are accessible at ROCplot.org.
