# Supplementary material for: Tumor CTR1 Expression and Systemic Copper Dynamics Converge on a Copper Axis in High-Grade Triple-Negative Breast Cancer
Source: Cancer Res Commun. 2026 Jun 30;6(6):1531–8. doi: 10.1158/2767-9764.CRC-26-0036 (PMC13316778; doi:10.1158/2767-9764.CRC-26-0036)
Supplement: Figure S1 — Baseline ceruloplasmin activity was measured across breast cancer molecular subtypes and healthy volunteers. [file crc-26-0036_figure_s1_suppsf1.pdf]

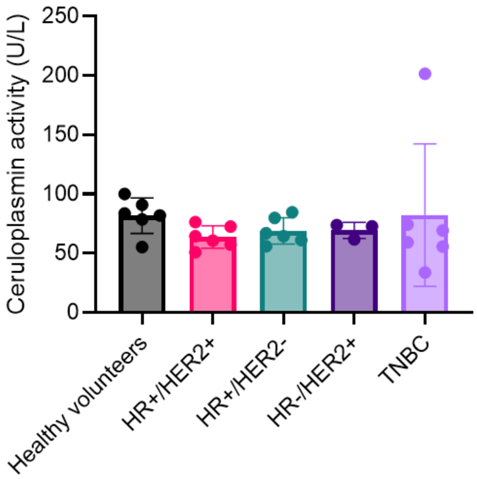

Figure S1. Baseline ceruloplasmin activity across breast cancer subtypes and healthy volunteers. Ceruloplasmin (CP) activity was measured in baseline serum samples from breast cancer patients in the prospective cohort and healthy volunteers. The cohort included HR+/HER2+ (n = 6), HR+/HER2− (n = 6), HR−/HER2+ (n = 3), and triple-negative breast cancer (TNBC; n = 6) patients, along with healthy volunteers (n = 6). CP activity was quantified using a colorimetric oxidase assay.
