# Supplementary material for: Tumor CTR1 Expression and Systemic Copper Dynamics Converge on a Copper Axis in High-Grade Triple-Negative Breast Cancer
Source: Cancer Res Commun. 2026 Jun 30;6(6):1531–8. doi: 10.1158/2767-9764.CRC-26-0036 (PMC13316778; doi:10.1158/2767-9764.CRC-26-0036)
Supplement: Figure S2 — This figure shows the relationship between baseline serum copper concentration and ceruloplasmin activity across breast cancer subtypes and healthy volunteers. [file crc-26-0036_figure_s2_suppfs2.pdf]

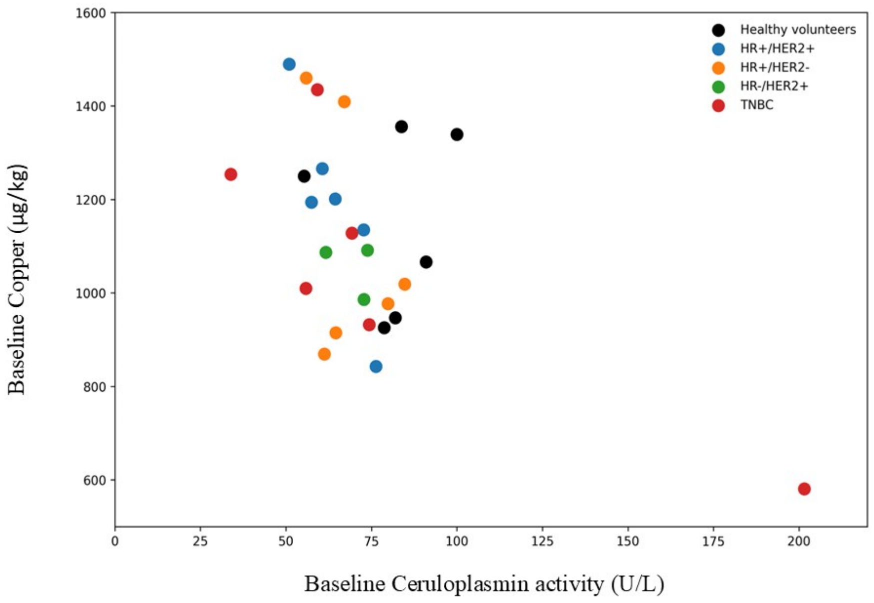

Figure S2. Relationship between baseline serum copper and ceruloplasmin activity across breast cancer subtypes and healthy volunteers. Scatter plot showing the relationship between baseline serum copper concentration and ceruloplasmin (CP) activity across healthy volunteers (n = 6) and breast cancer subtypes: HR+/HER2+ (n = 6), HR+/HER2− (n = 6), HR−/HER2+ (n = 3), and TNBC (n = 6). Each point represents an individual subject. This analysis evaluates whether baseline serum copper levels are associated with CP activity across the cohort.
