# Supplementary material for: Tumor CTR1 Expression and Systemic Copper Dynamics Converge on a Copper Axis in High-Grade Triple-Negative Breast Cancer
Source: Cancer Res Commun. 2026 Jun 30;6(6):1531–8. doi: 10.1158/2767-9764.CRC-26-0036 (PMC13316778; doi:10.1158/2767-9764.CRC-26-0036)
Supplement: Figure S3 — This figure compares therapy-associated changes in serum copper and ceruloplasmin activity across breast cancer molecular subtypes. [file crc-26-0036_figure_s3_suppsf3.pdf]

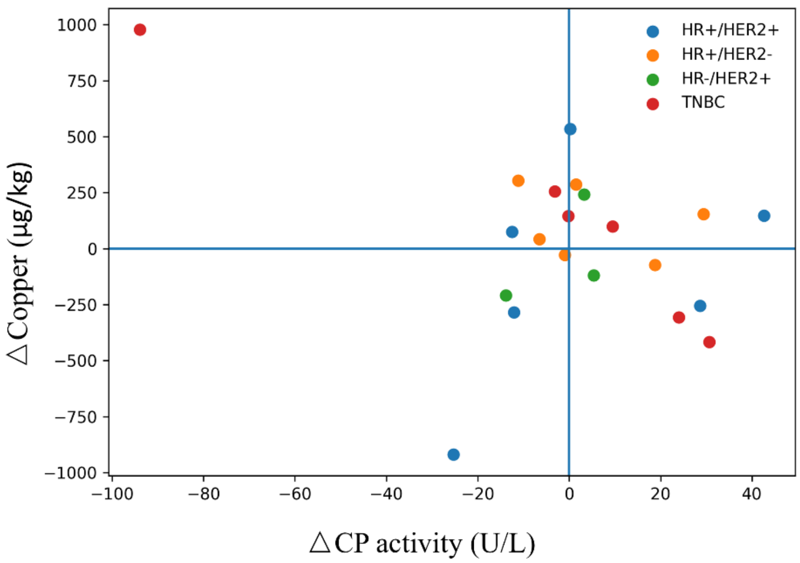

Figure S3. Relationship between therapy-associated changes in serum copper and ceruloplasmin activity across breast cancer subtypes. Scatter plot comparing therapy-associated changes in serum copper (Δcopper) and ceruloplasmin (ΔCP) activity in breast cancer patients following neoadjuvant therapy. Each point represents an individual patient across subtypes: HR+/HER2+ (n = 6), HR+/HER2− (n = 6), HR−/HER2+ (n = 3), and TNBC (n = 6). This analysis assesses whether changes in serum copper are concordant with changes in CP activity, thereby evaluating the contribution of ceruloplasmin-linked inflammatory responses to observed copper dynamics.
