# Supplementary material for: Tumor CTR1 Expression and Systemic Copper Dynamics Converge on a Copper Axis in High-Grade Triple-Negative Breast Cancer
Source: Cancer Res Commun. 2026 Jun 30;6(6):1531–8. doi: 10.1158/2767-9764.CRC-26-0036 (PMC13316778; doi:10.1158/2767-9764.CRC-26-0036)
Supplement: Figure S4 — This figure shows Kaplan–Meier survival analysis of grade 3 triple-negative breast cancer patients stratified by SLC31A1 expression. [file crc-26-0036_figure_s4_suppsf4.pdf]

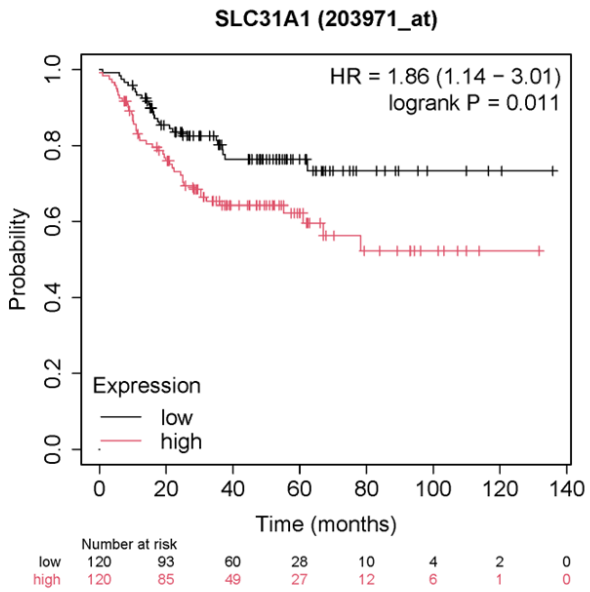

Figure S4. High *SLC31A1* expression is associated with reduced survival in grade 3 triple-negative breast cancer. Kaplan–Meier survival analysis of patients with grade 3 triple-negative breast cancer (TNBC) stratified by *SLC31A1* (CTR1) expression using the KMplotter platform. Patients were divided into high and low expression groups based on the optimal cutoff determined by the algorithm. Survival differences were evaluated using the log-rank test, and hazard ratios (HR) with corresponding P values are indicated. This analysis demonstrates the association between elevated tumor *SLC31A1* expression and poorer clinical outcomes in high-grade TNBC.
