# Supplementary material for: Tumor CTR1 Expression and Systemic Copper Dynamics Converge on a Copper Axis in High-Grade Triple-Negative Breast Cancer
Source: Cancer Res Commun. 2026 Jun 30;6(6):1531–8. doi: 10.1158/2767-9764.CRC-26-0036 (PMC13316778; doi:10.1158/2767-9764.CRC-26-0036)
Supplement: Figure S5 — This figure shows pan-cancer SLC31A1 expression in tumor versus normal tissues using TCGA data accessed through GEPIA. [file crc-26-0036_figure_s5_suppsf5.pdf]

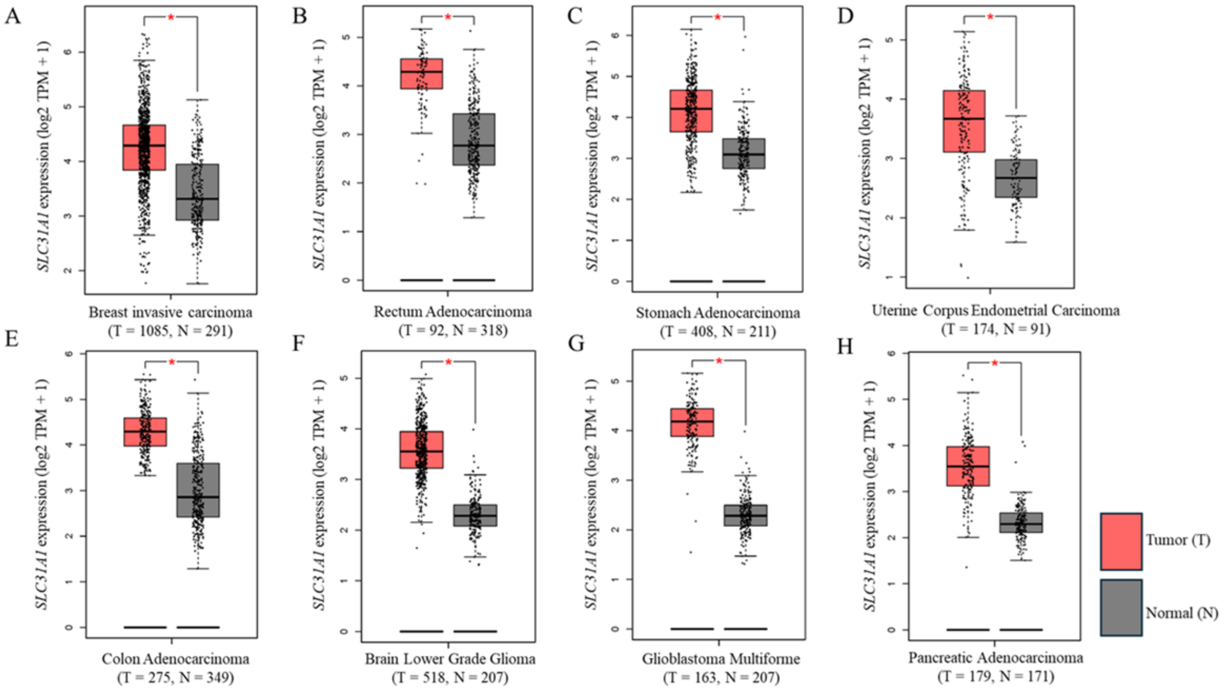

Figure S5. Pan-cancer analysis of *SLC31A1* expression in tumor versus normal tissues. *SLC31A1* (CTR1) expression levels were analyzed across multiple cancer types using transcriptomic data from The Cancer Genome Atlas (TCGA), accessed via the GEPIA platform. Gene expressions are presented as transcripts per million (TPM) on the y-axis. Expression in tumor tissues was compared with corresponding normal tissues where available. This analysis provides context for the baseline expression of *SLC31A1* across cancers and supports its broader relevance in tumor biology.
