# Supplementary material for: Tumor CTR1 Expression and Systemic Copper Dynamics Converge on a Copper Axis in High-Grade Triple-Negative Breast Cancer
Source: Cancer Res Commun. 2026 Jun 30;6(6):1531–8. doi: 10.1158/2767-9764.CRC-26-0036 (PMC13316778; doi:10.1158/2767-9764.CRC-26-0036)
Supplement: Table S1 — This table lists baseline serum copper and ceruloplasmin activity values for healthy volunteers and breast cancer patients grouped by molecular subtype. [file crc-26-0036_table_s1_suppst1.pdf]

Table S1: Baseline ceruloplasmin activity and serum copper values in healthy volunteers and breast cancer patients.

Subtypes	Patient #	Baseline Copper (µg/kg)	Baseline Ceruloplasmin activity (U/L)
HR+/HER2+	BCP-001	1266.00	60.66
	BCP-002	1135.00	72.70
	BCP-005	1194.00	57.48
	BCP-008	843.00	76.34
	BCP-020	1201.00	64.41
	BCP-021	1489.00	50.89
HR+/HER2-	BCP-006	977.00	79.86
	BCP-009	915.00	64.52
	BCP-012	869.00	61.23
	BCP-017	1460.00	55.89
	BCP-018	1409.00	67.02
	BCP-014	1019.00	84.75
HR-/HER2+	BCP-011	1091.00	73.84
	BCP-015	1087.00	61.68
	BCP-007	986.00	72.82
TNBC	BCP-003	581.00	201.53
	BCP-004	1128.00	69.30
	BCP-010	1254.00	33.85
	BCP-016	1435.00	59.19
	BCP-013	932.00	74.29
	BCP-019	1010.00	55.78
Healthy Volunteers	HV 39	1355.62	83.72
	HV 46	925.79	78.72
	HV 58	1249.91	55.32
	HV 05	1066.00	91.00
	HV 06	947.00	82.00
	HV 07	1339.00	100.00

Patient identifiers are anonymized and correspond to those presented in Table 1; patients are grouped here by molecular subtype.
