# Supplementary material for: Tumor CTR1 Expression and Systemic Copper Dynamics Converge on a Copper Axis in High-Grade Triple-Negative Breast Cancer
Source: Cancer Res Commun. 2026 Jun 30;6(6):1531–8. doi: 10.1158/2767-9764.CRC-26-0036 (PMC13316778; doi:10.1158/2767-9764.CRC-26-0036)
Supplement: Table S2 — This table lists therapy-associated changes in serum copper and ceruloplasmin activity for breast cancer patients grouped by molecular subtype. [file crc-26-0036_table_s2_suppst2.pdf]

Table S2: △Copper and △CP activity values in breast cancer patients

Subtypes	Patient #	△Copper (μg/kg)	△CP activity (U/L)
HR+/HER2+	BCP-001	148.00	42.60
	BCP-002	76.00	-12.50
	BCP-005	-255.00	28.63
	BCP-008	534.00	0.23
	BCP-020	-285.00	-12.04
	BCP-021	-919.00	-25.33
HR+/HER2-	BCP-006	-72.00	18.74
	BCP-009	43.00	-6.48
	BCP-012	286.00	1.48
	BCP-017	-28.00	-0.91
	BCP-018	154.00	29.42
	BCP-014	304.00	-11.13
HR-/HER2+	BCP-011	-209.00	-13.86
	BCP-015	242.00	3.29
	BCP-007	-119.00	5.34
TNBC	BCP-003	978.00	-93.95
	BCP-004	146.00	-0.23
	BCP-010	-416.00	30.67
	BCP-016	-307.00	23.97
	BCP-013	256.00	-3.18
	BCP-019	100.00	9.54

Patient identifiers are anonymized and correspond to those presented in Table 1; patients are grouped here by molecular subtype.
